# Modeling Human Liver Biology Using Stem Cell-Derived Hepatocytes

**DOI:** 10.3390/ijms141122011

**Published:** 2013-11-06

**Authors:** Pingnan Sun, Xiaoling Zhou, Sarah L. Farnworth, Arvind H. Patel, David C. Hay

**Affiliations:** 1 Shantou University Medical College, Shantou 515041, China; E-Mails: pingnan_sun@yahoo.com (P.S.); xiaolingsmiling_zhou@yahoo.com (X.Z.); 2 MRC Centre for Regenerative Medicine, University of Edinburgh, Edinburgh EH16 4UU, UK; E-Mail: slfarnworth@hotmail.com; 3 MRC-University of Glasgow Centre for Virus Research, University of Glasgow, Glasgow G11 5JR, UK; E-Mail: arvind.patel@glasgow.ac.uk

**Keywords:** drug metabolism, liver disease, stem cell, stem cell-derived hepatocytes, cell based models

## Abstract

Stem cell-derived hepatocytes represent promising models to study human liver biology and disease. This concise review discusses the recent progresses in the field, with a focus on human liver disease, drug metabolism and virus infection.

## 1. Introduction

The human liver has a multitude of functions. The cells which comprise the liver and perform those roles are termed parenchymal and non-parenchymal. Approximately 70%–80% of the liver is composed of the parenchymal cells known as hepatocytes. The non-parenchymal fraction make up the rest of the organ and are localized in the sinusoidal and biliary compartments of the tissue [1]. The hepatocyte compartment performs many metabolic and synthetic functions, for example storage and mobilization of sugars, maintenance of the blood clotting factors and the processing of drugs. Therefore, maintaining hepatic function is vital for normal physiology, and long-term damage to this compartment is detrimental to human health. Liver disease can be caused by a number of different stimuli, such as viral infection, excess alcohol or the exposure to prescription or recreational drugs. In the normal setting, the hepatocyte is capable of organ regeneration and restoration of normal function. However, in the chronic injury setting the residential stem cell population is required to restore liver mass and ultimately function [2].

Given the dire consequences of severe liver damage, a number of cell based models have been developed to predict the potential for human liver injury (for a review see [3]). Freshly isolated human primary hepatocytes (PHHs) are recognized as the “gold standard” for evaluating liver metabolism and drug toxicity [4]. PHHs express many of the essential drug metabolizing enzymes for several days in tissue culture following their isolation. However, enzyme expression drops off markedly during cell culture which can complicate biological interpretation [5,6]. In addition to their instability, PHHs widespread use is limited by their scarcity and quality [7].

**Figure 1:**
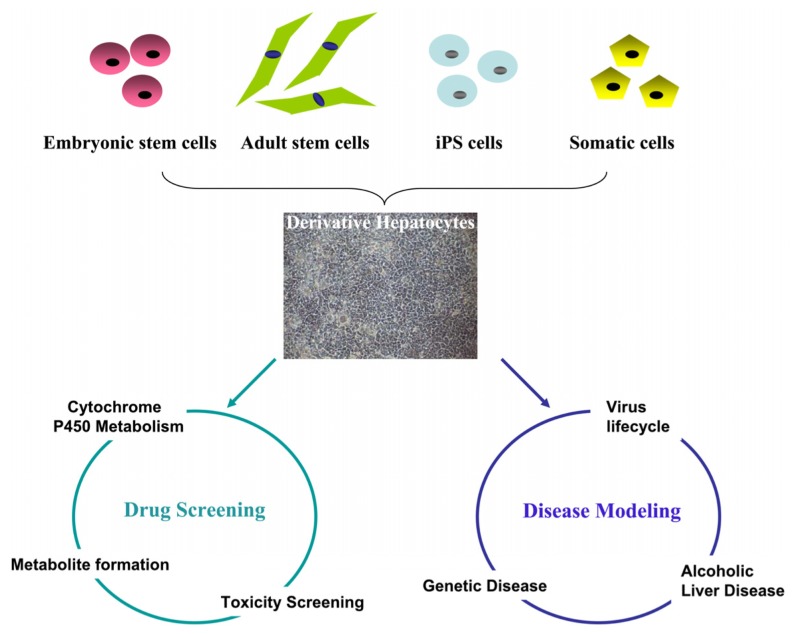
Modeling human liver biology using stem cell-derived hepatocytes. Hepatocytes derived from pluripotent stem cells (embryonic stem cells and induced pluripotent stem (iPS) cells), adult stem cells, or somatic cells can be used for modeling human liver biology “in a dish”. Examples of the use of this technology include, toxicity screening, disease modeling, viral infection and replication.

To overcome the shortage and stability of PHHs, a number of different human hepatoma-derived cell lines have been established. In contrast to PHHs, hepatoma-derived cells are scalable, cheap to maintain and are easy to handle. However, those benefits are out-weighed by defects in key cell signaling pathways which result in inferior phenotype. For example, Huh7.5 cells are used to study Hepatitis C virus (HCV) replication but are not ideal to study virus-host interaction due to defects in the retinoic acid-inducible gene 1 pathway [8]. Additionally, hepatoma-derived cell lines display limited capacity for drug metabolism due to poor cytochrome *P450* gene expression [9–11]. With the advent of pluripotent stem cell technology, it is now possible to produce stem cell-derived hepatocyes at scale and from known genetic background. We believe that this has revolutionary potential for modern medicine (Figure 1) and discuss the potential of stem cells in more detail throughout the review.

## 2. Generation of *In Vitro*-Derived Hepatocytes from Stem Cell or Somatic Cells

### 2.1. Hepatocytes Derived from Human Embryonic Stem Cells (hESCs)

hESCs are derived from the inner cell mass of preimplantation embryos [12]. They have the ability to self-renew, and at the same time retain the ability to differentiate to all three germ layers [13]. Therefore, it is possible to scale large numbers of stem cells and their derivatives for downstream application [14]. hESCs have been differentiated into functional hepatic endoderm using spontaneous or directed differentiation [15–18]. During spontaneous differentiation, hESCs are maintained in suspension to induce differentiation into embryoid bodies (EBs) [19]. Those EBs are further stimulated to differentiate toward the hepatocyte lineage using physiological cues [20]. While the process is resilient, the efficiency of stem cell-derived hepatocytes generated using this method is low and requires further enrichment [16,21]. During directed differentiation, hESCs are differentiated to hepatocytes in adherent and 2 dimensional monolayer culture. Several groups have confirmed this method is possible and more efficient than spontaneous differentiation [17,22,23]. In both procedures, definitive endoderm is specified from hESCs and then induced to differentiate along the hepatic lineage by the sequential addition of inducing factors. We have developed a highly efficient method to deliver functional hepatocytes which could be assayed in a high throughput format [18]. Since those early discoveries, we and others have delivered scalable populations of stem cell-derived hepatocytes using serum-free and synthetic components [24,25]. Most recently, exciting studies by Vosough *et al*. demonstrated scalable suspension culture for stem cell expansion and differentiation. Most notably, pluripotent stem cells were expanded and differentiated toward the hepatocyte lineage at a scale that would satisfy liver cell-based therapy requirements [21,26].

### 2.2. Hepatocytes Derived from Induced Pluripotent Stem Cells (iPSCs)

Differentiated somatic cells can be reprogrammed to an ES-like state [27] termed induced pluripotent stem cells (iPSCs). As with the hESCs, iPSCs theoretically offer a potential source of somatic cells in large numbers [28]. Of note, iPSCs have been induced toward the hepatocyte lineage using similar protocols that were developed in hESCs [29]. Interestingly and in agreement with studies using hESCs, there are significant variations in iPSC hepatic differentiation capacity [29]. iPSC clones derived from peripheral blood cells were found to be comparable to dermal fibroblasts from the same individual, but differentiation capacity varied from donor to donor using a modified version of our procedure [18,30].

### 2.3. Hepatocytes from Direct Reprogramming Somatic Cells

Reprogramming of fibroblasts to iPSC demonstrated that somatic cells could be reprogrammed to a pluripotent stem cell state [31,32]. It has also been demonstrated that somatic cells are capable of trans-differentiation to heptocytes using specific sub sets of transcription factors. Murine somatic cells have been successfully reprogrammed to hepatocyte-like cells [33,34]. Hepatocytes were derived from mouse tail-tip fibroblasts and transduced with virus expressing Gata4, HNF1α and Foxa3 [30]. In these studies there was a requirement for the inactivation of p19 (Arf) [33]. In a separate study, mouse embryonic and adult fibroblasts were trans-differentiated using combinations of HNF4α plus Foxa1, Foxa2 or Foxa3 [34]. Importantly, the derivative hepatocytes exhibited similar function to primary hepatocytes. Encouragingly, trans-differentiated heaptocytes were transplanted *in vivo* and repopulated the livers of fumarylacetoacetate-hydrolase-deficient mice, rescuing almost half of recipients [33]. Most recently, the transcription factor cocktail was modified to HNF1β and Foxa3, which yielded a cell type reminiscent of bipotent hepatic stem cells [35]. Until now, the direct reprogramming to hepatocytes has not been accomplished in human cells, but this cannot be far off. In support of this, over expression of lineage-specific transcription factors has been shown to directly convert terminally differentiated cells into other lineages, including neurons, cardiomyocytes and blood progenitors [36–38].

## 3. Modeling Human Drug Metabolism Using Stem Cell-Derived Hepatocytes

### 3.1. Drug Attrition

The drug discovery process is a rocky road and pharmaceutical companies face major issues with drug attrition [39]. It is estimated that the average cost to bring a new drug to market ranges between $800 million and $2 billion [40]. Issues associated with cardiac and liver toxicity, among others, are a major concern during this process [41]. Therefore, human models, which accurately reflect physiology, could impact on the spiraling costs associated with drug development. As previously detailed, PHHs represent the “gold standard” [42], but their limited availability and stability restricts widespread use. A credible alternative for the future, are pluripotent stem cell-derived hepatocytes, which have already shown great potential in the determination of human drug metabolism and hepatotoxicity [43].

### 3.2. Stem Cell-Derived Hepatocytes

Drug metabolism in the liver can be divided into three phases. Phase I reactions are carried out by the cytochrome P450 enzymes (CYPs) which modify the chemical substrate via oxidation, reduction and/or hydrolysis. CYPs are the major enzymes involved in drug metabolism [44]. The major CYP enzymes involved in drug metabolism include; the CYP1A family, CYP2A6, CYP2B6, the CYP2C family, CYP2D6, CYP2E1, and the CYP3A family [45]. Among them, CYP3A4 is the most abundant isoform in human liver and has been estimated to be involved in the metabolism of approximately 50% of prescribed medicines [46]. Phase II reactions are conjugation reactions which modify drug and metabolite polarity, rendering the products more water soluble. Following this, the Phase III reactions are responsible for metabolite transport across the cell membrane. In this stage, transporters, such as multi-drug resistance-associated protein 2 and permeability glycoprotein, move metabolites across the cell barriers in an energy dependent process [47]. Phase I–III reactions are vital to normal hepatocyte function. Unfortunately, drug metabolism, conjugation and transport decrease with time in cell culture and has been attributed to sub-optimal tissue culture microenvironments [48]. Recently, our laboratory identified a synthetic polymer which delivers phenotypically stable hepatocyte populations for at least 15 days, in two dimensional and three-dimensional format. Importantly, the stem cell derived heaptocytes replated on this material displayed equivalence to the current gold standard demanded by regulatory authorities [25,43]. In addition to our approach, other groups have also studied human liver toxicity using stem cell derived hepatocytes. In a recent study by Yildirimman *et al*., stem cell-derived hepatocytes were exposed to numerous agents classified as noncarcinogens, genotoxic carcinogens, and nongenotoxic carcinogens [49]. Changes in hepatic transcriptome were analyzed using gene expression microarrays. Encouragingly, stem cell-derived hepatocytes correlated well with that of PHHs.

## 4. Modeling Human Disease Using Stem Cell-Derived Hepatocytes

### 4.1. Modeling Human Genetic Liver Disorders

Stem cell-derived hepatocytes generated from patients with metabolic liver diseases are a useful model for understanding the disease process. Various human inherited liver diseases such as: a1-antitrypsin deficiency; Glucose-6-phosphate deficiency; LDL-receptor mutations; Crigler-Najjar-Syndrome; and Wilson’s disease have been successfully modeled “in a dish” using iPS-derived hepatocytes from the patients [50–52]. In addition to understanding disease better, these models also provide useful platforms to screen for new drugs to better treat disease and therefore represent exciting advances.

### 4.2. Hepatitis Virus Infection and Replication

In addition to modeling inherited liver diseases, stem cell-derived hepatocytes have proved useful in delivering models for studying the lifecycle of hepatotropic viruses. This is very important as current approaches using human hepatoma cell culture to model hepatitis C virus (HCV) lifecycle has a number of drawbacks and as a result does not support the growth of clinical viral isolates. This precludes studies of many important host-pathogen interactions that are crucial for a more detailed understanding of viral life cycle and pathogenesis. This has necessitated the development of models which are phenotypically closer to human hepatocytes. Encouragingly, stem cell-derived hepatocytes have been shown to support the entire life cycle of the HCV including viral entry, replication, production and release of progeny virus, albeit at low levels [53–55]. However, there are still few reports on applying stem cell-derived hepatocytes to other hepatotropic viruses including hepatitis B virus (HBV) and hepatitis D virus (HDV) which affect more than 2 billion people worldwide [56–58]. A suitable model for HBV, and the associated, HDV are needed to provide key clues to viral lifecycle and pathogenesis. Of note, sodium taurocholate, a transport polypeptide, was recently discovered as a new functional receptor of HBV and HDV [59] and represents substantial progress in this field. We believe that stem cell-derived hepatocytes offer promising models for HBV and HDV studies.

Another important goal of developing such virus models, is to further our understanding of the affect that genetic background has on viral pathogenesis. Certain individuals are prone to developing chronic viral infection while others can efficiently clear the virus. A reasonable explanation may lie in differences in immune factors or viral receptor expression. However, the true reasons for these different responses are still largely unresloved. We believe that stem cell derived models, from defined genetic origin, may provide insight into the disease process and currently available treatments. In the future, such models could facilitate the development of new anti-viral drugs, which can be tailored to the patient’s genetics.

### 4.3. Alcoholic Liver Disease

Liver disease, such as fatty liver, cirrhosis, and hepatocellular carcinoma are closely associated with alcohol abuse [60]. To investigate the mechanisms by which alcohol affects liver biology, many laboratories have used animal models, histology, primary human hepatocytes and hepatoma cell lines [61]. However, these models do not accurately reflect the situation within human liver as alcoholic liver disease pre-disposition is largely dependent on the individual’s genetics.

Human hepatocytes, are the major cell type in the liver which undertakes alcohol metabolism. Hepatocytes degrade alcohol into aldehyde by alcohol dehydrogenase and catalyze the oxidation of aldehydes to carboxylic acids by aldehyde dehydrogenase. In addition to tissue damage, alcohol and its metabolites, can trigger the innate immune response, contributing to inflammation [3,62]. Most recently, stem cell derived heaptocytes have been shown to have an important role to play in modeling human hepatocyte response to alcohol. Alcohol was found to induce apoptosis and disturb stem cell-derived hepatocyte differentiation as well as impair the survival and proliferation of stem cell-derived hepatocytes [63]. This has raised the possibility that by applying stem cell-derived hepatocytes from defined genetic origin, in a context of ALD, will allow the more detailed analysis of an individual’s response to alcohol metabolism and disease progression.

## 5. Challenge and Prospect of Stem Cell-Derived Hepatocytes

Many methods have been devised to efficiently generate stem cell-derived hepatocytes from hESCs or hiPSCs (for a review see Szkolnicka *et al*. 2013 [64]). There are still challenges to be faced when using stem cell-derived hepatocytes to model human liver physiology. Those challenges include, bona fide differentiation, cost effective scale up and reliable and stable performance. That being said, mature cell populations are emerging which deliver reliable biological readouts and represent exciting advances in the field, likely to impact on modern medicine [65].
